# Outcomes of conduction system pacing compared to right ventricular pacing as a primary strategy for treating bradyarrhythmia: systematic review and meta-analysis

**DOI:** 10.1007/s00392-021-01927-7

**Published:** 2021-08-19

**Authors:** Amr Abdin, Suleman Aktaa, Davor Vukadinović, Elena Arbelo, Harran Burri, Michael Glikson, Christian Meyer, Theresa Munyombwe, Jens Cosedis Nielsen, Christian Ukena, Kevin Vernooy, Chris P. Gale

**Affiliations:** 1 Cardiology, Angiology and Intensive Care Medicine, Internal Medicine Clinic III, Saarland University Hospital, Kirrberger Street 100, 66421 Homburg, Saarland Germany; 2 Leeds Institute of Cardiovascular and Metabolic Medicine, Faculty of Medicine and Health, University of Leeds, Leeds, LS2 9JT UK; 3 Department of Cardiology, Leeds Teaching Hospitals NHS Trust, Leeds, LS1 3EX UK; 4 Cardiology Department, Arrhythmia Section, Hospital Clínic, Universitat de Barcelona, C. Villarroel 170, Esc 3, Planta 6, 08036 Barcelona, Spain; 5 IDIBAPS, Institut D’Investigació August Pi I Sunyer (IDIBAPS), Barcelona, Spain; 6 Centro de Investigación Biomédica en Red de Enfermedades Cardiovasculares (CIBERCV), Madrid, Spain; 7 Cardiology Department, Geneva University Hospital, Geneva, Switzerland; 8 Cardiology Department, Shaare Zedek Hospital, affiliated to the Hebrew University, Jerusalem, Israel; 9 University Heart Center, Hamburg, Cardiac Neuro- and Electrophysiology Research Consortium, EVK Düsseldorf, Düsseldorf, Germany; 10 Department of Cardiology, Aarhus University Hospital, Aarhus, Denmark; 11 Department of Clinical Medicine, Aarhus University, Aarhus, Denmark; 12 Department of Cardiology, Cardiovascular Research Institute Maastricht (CARIM), Maastricht University Medical Center, Maastricht, The Netherlands; 13 Radboud University Medical Center, Nijmegen, The Netherlands

**Keywords:** Cardiac pacing, His‐bundle pacing, Left bundle branch pacing, Clinical outcomes, Meta-analysis, Systematic review

## Abstract

**Background:**

Right ventricular pacing (RVP) may cause electrical and mechanical desynchrony leading to impaired left ventricular ejection fraction (LVEF). We investigated the outcomes of RVP with His bundle pacing (HBP) and left bundle branch pacing (LBBP) for patients requiring a de novo permanent pacemaker (PPM) for bradyarrhythmia.

**Methods and results:**

Systematic review of randomized clinical trials and observational studies comparing HBP or LBP with RVP for de novo PPM implantation between 01 January 2013 and 17 November 2020 was performed. Random and fixed effects meta-analyses of the effect of pacing technology on outcomes were performed. Study outcomes included all-cause mortality, heart failure hospitalization (HFH), LVEF, QRS duration, lead revision, atrial fibrillation, procedure parameters, and pacing metrics. Overall, 9 studies were included (6 observational, 3 randomised). HBP compared with RVP was associated with decreased HFH (risk ratio [RR] 0.68, 95% confidence interval [CI] 0.49–0.94), preservation of LVEF (mean difference [MD] 0.81, 95% CI − 1.23 to 2.85 vs. − 5.72, 95% CI − 7.64 to -3.79), increased procedure duration (MD 15.17 min, 95% CI 11.30–19.04), and increased lead revisions (RR 5.83, 95% CI 2.17–15.70, *p* = 0.0005). LBBP compared with RVP was associated with shorter paced QRS durations (MD 5.6 ms, 95% CI − 6.4 to 17.6) vs. (51.0 ms, 95% CI 39.2–62.9) and increased procedure duration (MD 37.78 min, 95% CI 20.04–55.51).

**Conclusion:**

Of the limited studies published, this meta-analysis found that HBP and LBBP were superior to RVP in maintaining physiological ventricular activation as an initial pacing strategy.

**Supplementary Information:**

The online version contains supplementary material available at 10.1007/s00392-021-01927-7.

## Introduction

Permanent pacing with right ventricular stimulation is frequently used in patients with bradyarrhythmia, and recommended by current international guidelines because it is associated with improved clinical outcomes [1]. The extent of right ventricular pacing (RVP) varies between patients and many individuals tolerate a high proportion of RVP without complications [2–6]. However, chronic RVP may cause electrical and mechanical desynchrony leading to impaired left ventricular ejection fraction (LVEF), mitral and tricuspid valve regurgitation and an increased risk of atrial fibrillation (AF) [2–7]. While the benefits of biventricular pacing (BiVP) over RVP in patients with reduced LVEF and atrioventricular (AV) block, who require ventricular pacing, is established [8], the optimal pacing strategy for those with normal or mildly reduced LVEF is less well understood.

Novel pacing technologies, such as His bundle pacing (HBP) and left bundle branch pacing (LBBP), have emerged to maintain physiological ventricular activation via the native His‐Purkinje system [9–12]. However, there is limited information concerning the comparative effectiveness of those novel pacing strategies against RVP in patients with normal or mildly reduced LVEF [10, 11] and thus the optimal pacing method for this group of patients remains uncertain. Notably, both American and European guidelines recommend RVP as an initial pacing strategy for patients with normal or mildly reduced LVEF [1, 7]. Therefore, we aimed to compare HBP and LBBP with RVP as an initial pacing strategy for patients requiring de novo permanent pacemaker implantation for bradyarrhythmia. This work was initiated after questions emerging during the development of the cardiac pacing quality indicators (QIs) for the 2021 European Society of Cardiology (ESC) Clinical Practice Guidelines on cardiac pacing and cardiac resynchronization therapy [1].

## Methods

### Systematic review

#### Search strategy

We conducted a systematic review of the published randomized controlled trials (RCTs) and controlled observational studies in accordance with the Preferred Reporting Items for Systematic Review and Meta-Analyses (PRISMA) statement [14], using MEDLINE and Embase via OVID_@._ The initial search strategy was developed in MEDLINE using keywords and Medical Subject Headings (MeSH) terms (Supplementary material, Table S1), and the final strategies were then developed using an iterative process incorporating findings from citations and grey literature search. We included the main publications of major studies from which our search obtained only sub-studies. The search was restricted to full-text articles published in English between 01 January 2013 and 17 November 2020. The year 2013 was selected because it corresponds to the publication of the last ESC Clinical Practice Guidelines on cardiac pacing and cardiac resynchronisation therapy [1].

#### Study selection

We included studies that: [1] compared directly the effects of HBP or LBBP versus RVP, [2] evaluated adults (≥ 18 years of age) with bradyarrhythmia and an indication for de novo permanent pacing, [3] reported at least one outcome of interest for comparison at implantation and at any point during the follow-up period, and [4] provided data that allowed the comparison between the study arms (i.e. means and standard deviations [SD] or medians and interquartile ranges [IQR]). When data by the same authors or the same institution in an overlapping period were identified, only the most recent results were considered. A reference manager software (Zotero) was used for duplicates removal and data management. Two reviewers (AA and SA) independently reviewed the abstracts of the identified articles against the predefined inclusion criteria. Disagreements were solved with discussion.

#### Data extraction

For the selected studies, two investigators (AA and SA) reviewed the full texts and used the same template to extract data relevant to the analysis on an Excel spreadsheet. The study design, as well as the sample size, pacing characteristics, duration of follow-up and primary endpoints were extracted as shown in Table1.

**Table 1:** Baseline characteristics of studies comparing, HBP, LBBP, and RVP as a primary pacing strategy

Study	Study design	FU (months)	Pacing mode	Number of participants	Indication for pacing	Primary endpoint	Baseline EF (%)	Pacing burden %
Catanzariti et al. 2013 [15]	Observtional crossover	34.6	HPB vs. RVP	26 vs. 26	AV conduction disease or SND	LV dyssynchrony and function	57.2 + 7.4	NA
Kronborg et al. 2014 [16]	RCT crossover	12	HPB vs. RVP	19 vs. 19	AV block	LVEF	56 ± 10 vs. 55 ± 7	> 99
Pastore et al. 2016 [18]	retrospective	58	HPB vs. RVP	148 vs. 329	AV block	AF occurrence	62 + 7 vs 60 + 8	NA
Vijayaraman et al. 2017 [17]	retrospective	60	HPB vs. RVP	94 vs. 98	AV conduction disease or SND	Safety and success rate of HBP	55 ± 8 vs 57 ± 7	59 ± 43 vs 57 ± 45
Abdelrahman et al. 2018 [12]	Prospective non-randomized	24	HPB vs. RVP	332 vs.433	AV conduction disease or SND	death, HFH or upgrade to BiVP	54.9 ± 8.5 vs 54.2 ± 10.2	54–58 for both groups
Wang et al. 2019 [20]	Prospective- randomized	6	LBBP vs. RVP	66 vs. 65	AV conduction disease or SND	Depolarization-repolarization indices	61.3 ± 5.7 vs 62.1 ± 6.3	NA
Zhang et al. 2019 [19]	Prospective- randomized	0	LBBP vs. RVP	20 vs. 21	AV conduction disease or SND	Immediate clinical outcomes	45.7 ± 18.4 vs 65.9 ± 4.1	NA
Chen et al. 2019 [22]	Prospective non-randomized	3	LBBP vs. RVP	20 vs. 20	AV conduction disease or SND	ECG and pacing characteristics	60 ± 10.6 vs 60.7 ± 6	NA
Cai et al. 2019 [21]	Prospective non-randomized	0	LBBP vs. RVP	40 vs. 38	SND	Electrical and mechanical synchrony	> 53 in both groups	NA

Baseline characteristics of studies comparing, HBP, LBBP, and RVP as a primary pacing strategy for patients required permanent pacemaker

*AF* atrial fibrillation, *AV* atrioventricular, *BiVP* biventricular pacing, *ECG* electrocardiogram, *EF* ejection fraction, *FU* follow-up, *HBP* his bundle pacing, *HFH* heart failure hospitalization, *LBBP* left bundle branch pacing, *LVEF* left ventricular ejection fraction, *LVESV* left ventricular end-systolic volume, *NA* not available, *RCT* randomized control trial, *RVP* right ventricular pacing, *SND* sick node disease

#### Appraising the quality of the review studies

Cochrane Risk of Bias Assessment was used to evaluate the quality of RCT (Supplementary material, Table S2), and Newcastle–Ottawa Scale Assessment for cohort studies (Supplementary material, Table S3). Due to the small number of included trials (< 5) for each comparison group, exploration of any potential publication bias was not performed.

### Statistical analysis

The primary outcomes for the study were mortality and heart failure hospitalization (HFH). The secondary outcomes were changes in LVEF, AF occurrence, paced QRS (pQRS) complex duration, procedure duration, lead revision rates and pacing threshold. To compare outcomes between studies investigating RVP (control group) with studies investigating HBP and LBBP, we pooled the available data (number of events for dichotomous variables, and average value, standard deviation and sample size for continuous variables) for each outcome of interest from the included studies. Differences in events rates and average values for specific outcome among groups were determined and presented using Forest plots with corresponding 95% confidence intervals (CI) for each study. The effect measure for dichotomous variables was quantified as risk ratios (RR), and for continuous variables was the mean difference (MD). Meta-analysis was conducted and the data from each study were pooled using fixed (Mantel–Haenszel, Rothman-Boice) or random effects (DerSimonian-Laird) model, as appropriate. Statistical heterogeneity between the trials was assessed using Cochran’s Q test and Higgins *I*^2^ statistic. Relevant statistical heterogeneity was present in cases where Cochran’s Q test *p* < 0.05 and *I*^2^ > 50%, for which cases we used random-effects models. All statistical analyses were conducted using RevMan 5.3 software. All *p* values were two-sided, with *p* < 0.05 considered as significant.

## Results

**Fig. 1:**
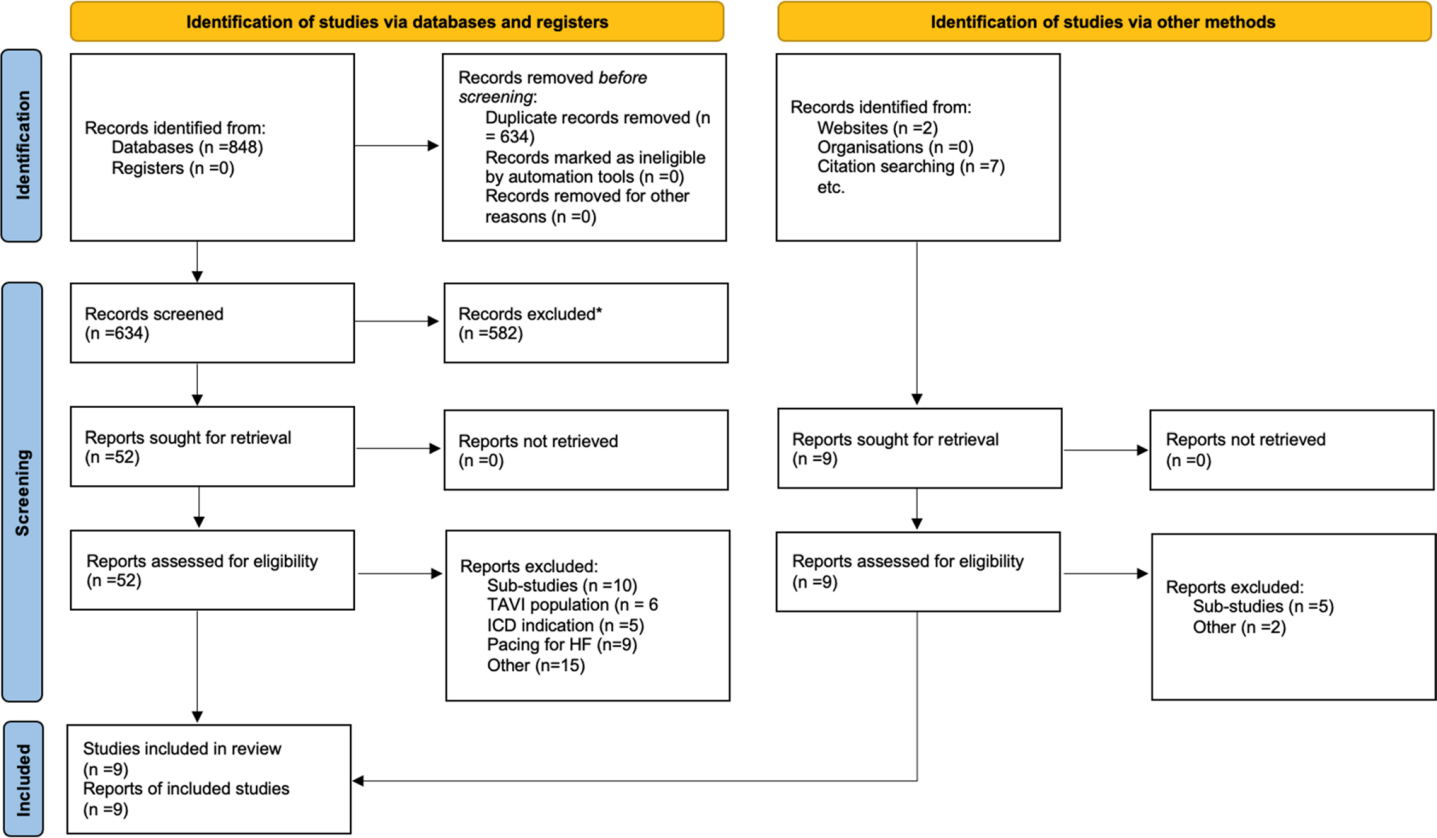
PRISMA flowchart for the studies included and reasons for studies excluded from the systematic review. *HF* heart failure, *ICD* implantable cardioverter defibrillator, *TAVI* transcatheter aortic valve implantation

In total, 848 studies were identified from the systematic review and an additional 7 were found by references review of the included articles. After the removal of duplicates, 641 studies remained and were evaluated against the predefined inclusion criteria. Of those, 53 studies were included for full-text review, and a further 46 studies were excluded leaving 9 studies (7 from the databases search) for the systematic review and meta-analysis (Fig. 1). Of the 9 studies, 5 compared HBP with RVP (*n* = 619 vs. *n* = 905 patients) with follow-up durations between 12 and 60 months [12, 15–18] and 4 compared LBBP with RVP (*n* = 149 vs. *n* = 144 patients) with follow-up durations between 3 and 6 months [19–22]. The pacing indication was sinus node disease (SND) in 1 study, AV conduction disease in 2 study and SND and AV conduction disease in 6 studies. The characteristics of the studies are summarized in Table 1.

Result of the pairwise comparisons against RVP analysed as a single entity are detailed below:

### HBP compared with RVP

#### Mortality and HFH

**Fig. 2:**
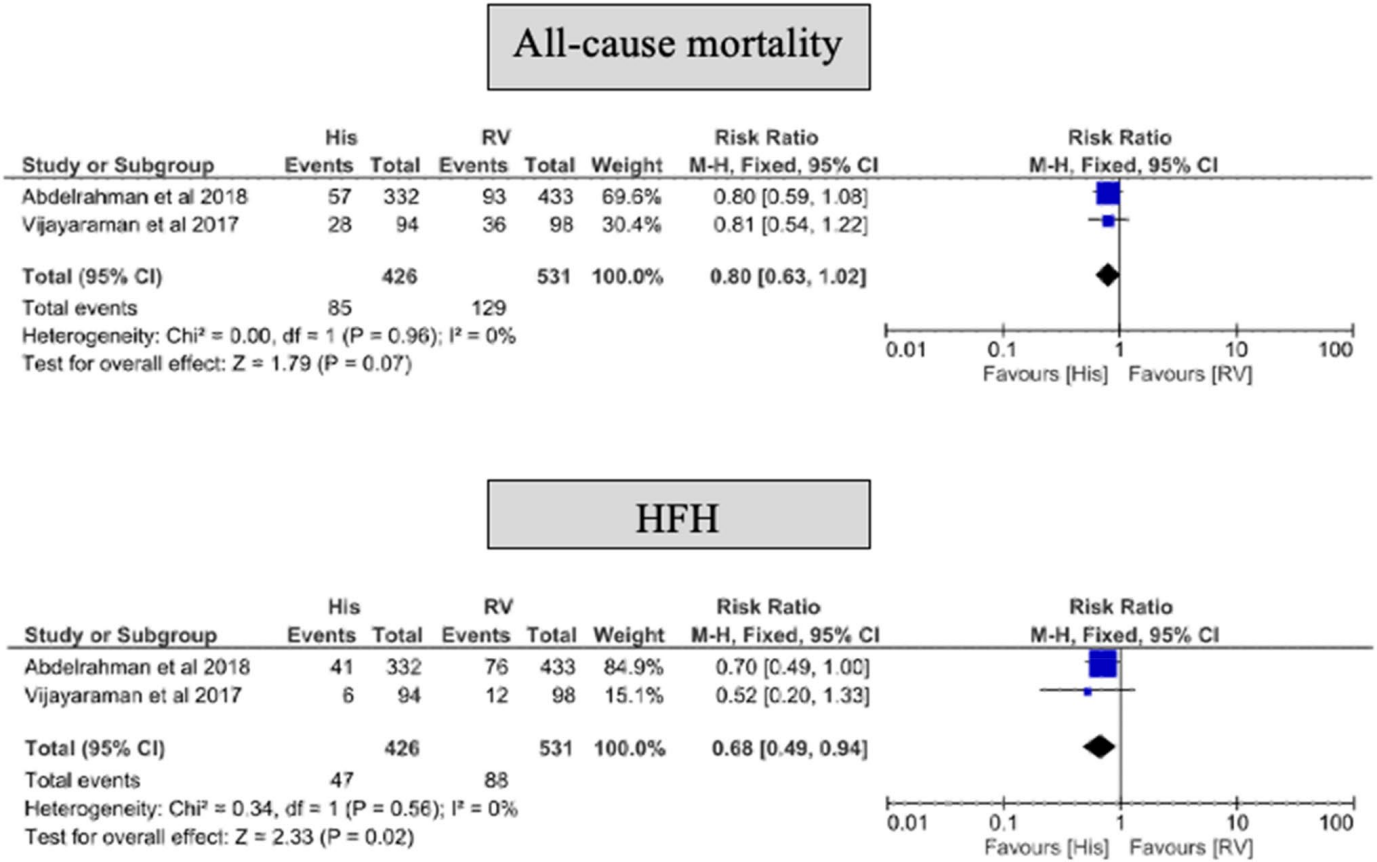
Forest plot of all‐cause mortality and HFH with HBP vs RVP for patients requiring permanent transvenous pacing after a 24-60 months follow-up. *CI* confidence interval, *HBP* His-bundle pacing, *HFH* heart failure hospitalization, *RR* risk reduction, *RVP* right ventricular pacing

Compared with RVP, HBP was associated with a decreased risk of HFH (RR: 0.68, 95% CI 0.49–0.94, *p* = 0.02) and no statistically significant difference in all-cause mortality compared with RVP (RR: 0.80, 95% CI 0.63–1.02, *p* = 0.07) (Fig. 2).

#### LVEF

**Fig. 3:**
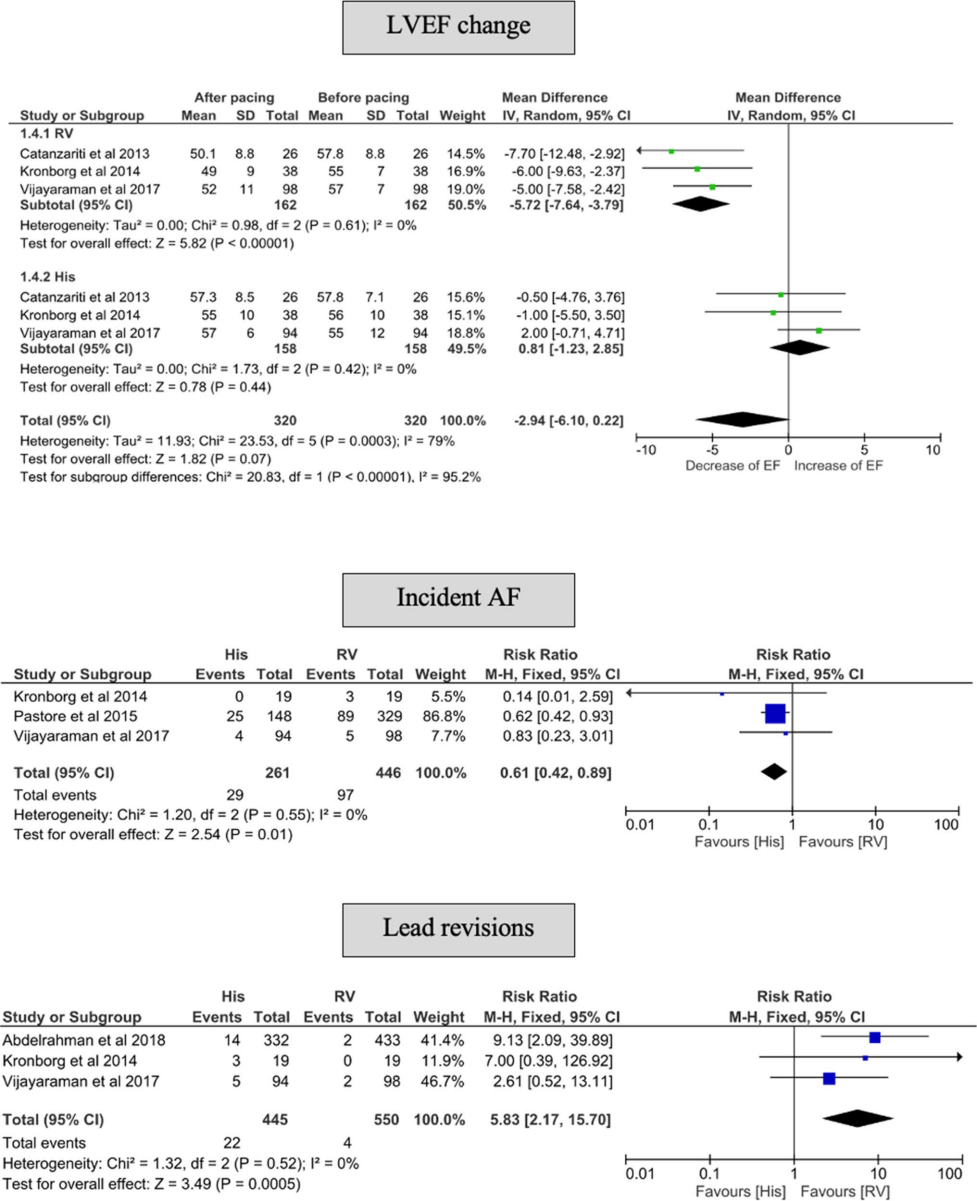
Secondary outcomes for HBP vs. RVP after 12–60 months follow‐up. LVEF change from baseline, new-onset AF and lead revisions. *AF* atrial fibrillation, *CI* confidence interval, *HBP* His-bundle pacing, *LVEF* left ventricular ejection fraction, *RR* risk reduction, *RVP* right ventricular pacing

In studies that reported the change in LVEF [15–17], a decrease in LVEF was associated with RVP (mean difference (MD) − 5.72, 95% CI − 7.64 to − 3.79, *p* = < 0.001) but not for HBP (MD 0.81, 95% CI − 1.23 to 2.85, *p* = 0.44) (Fig. 3), and there was a statistically significant interaction between RVP and HBP concerning their effects on LVEF (*p* for interaction < 0.001).

#### Incident AF

Compared with RVP, HBP was associated with a decreased risk of new-onset AF (RR 0.61, 95% CI 0.42–0.89, *p* = 0.01) (Fig. 3).

#### pQRS duration

**Fig. 4:**
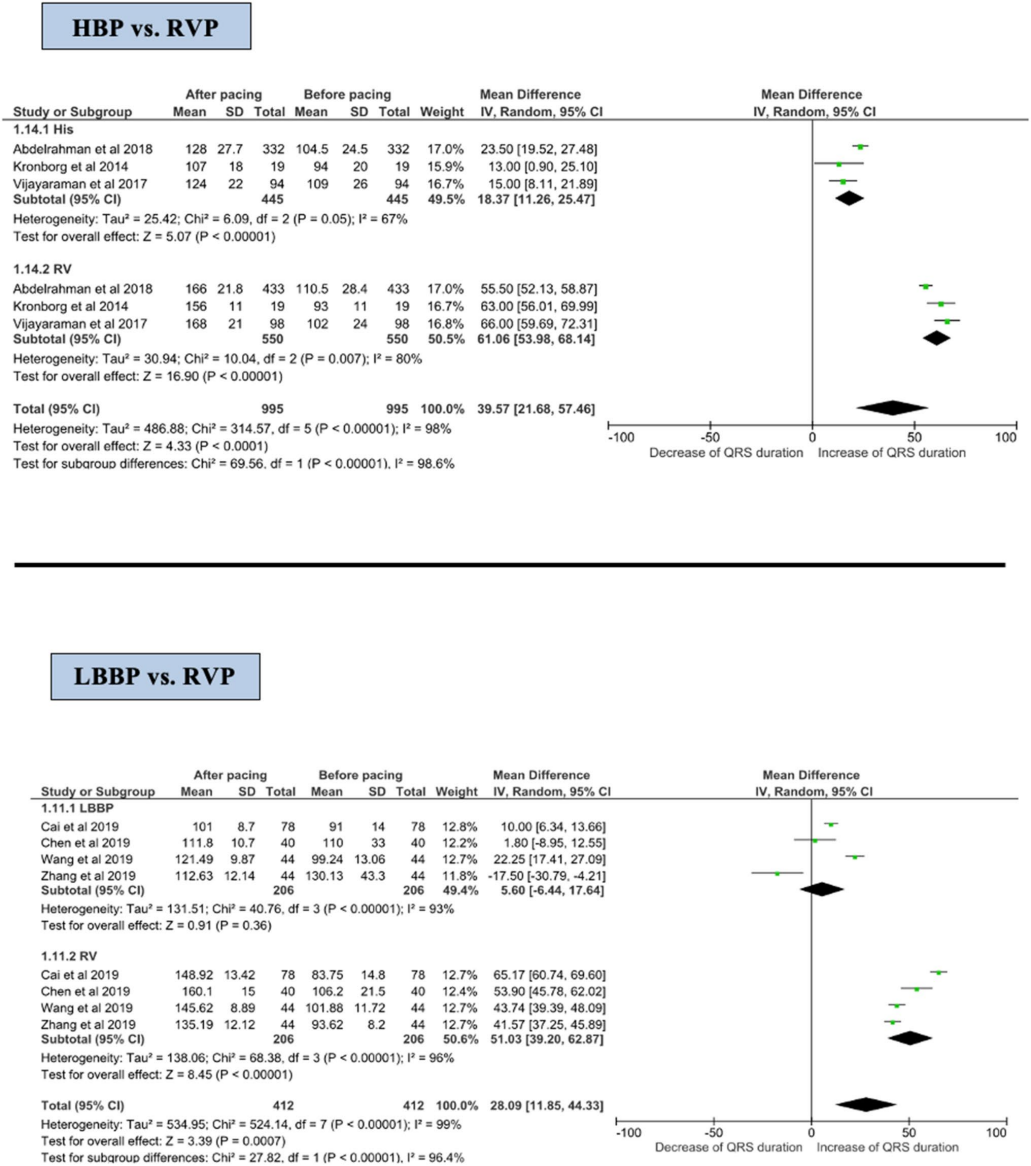
Forest plot of change in QRS duration before and after implantation among HBP and LBBP groups. *CI* confidence interval, *HBP* His-bundle pacing, *LBBP* left bundle branch pacing, *RR* risk reduction, *RVP* right ventricular pacing

The pQRS duration was longer in the RVP group compared with the HBP group (MD of 61.06 ms, 95% CI 53.98–68.14 ms) vs. 18.37 ms (95% CI 11.26–25.47 ms), respectively (Fig. 4). This was also reflected as a significant difference in the test for subgroup difference between HBP and RVP (*p* < 0.001).

#### Procedure and fluoroscopy duration

**Fig. 5:**
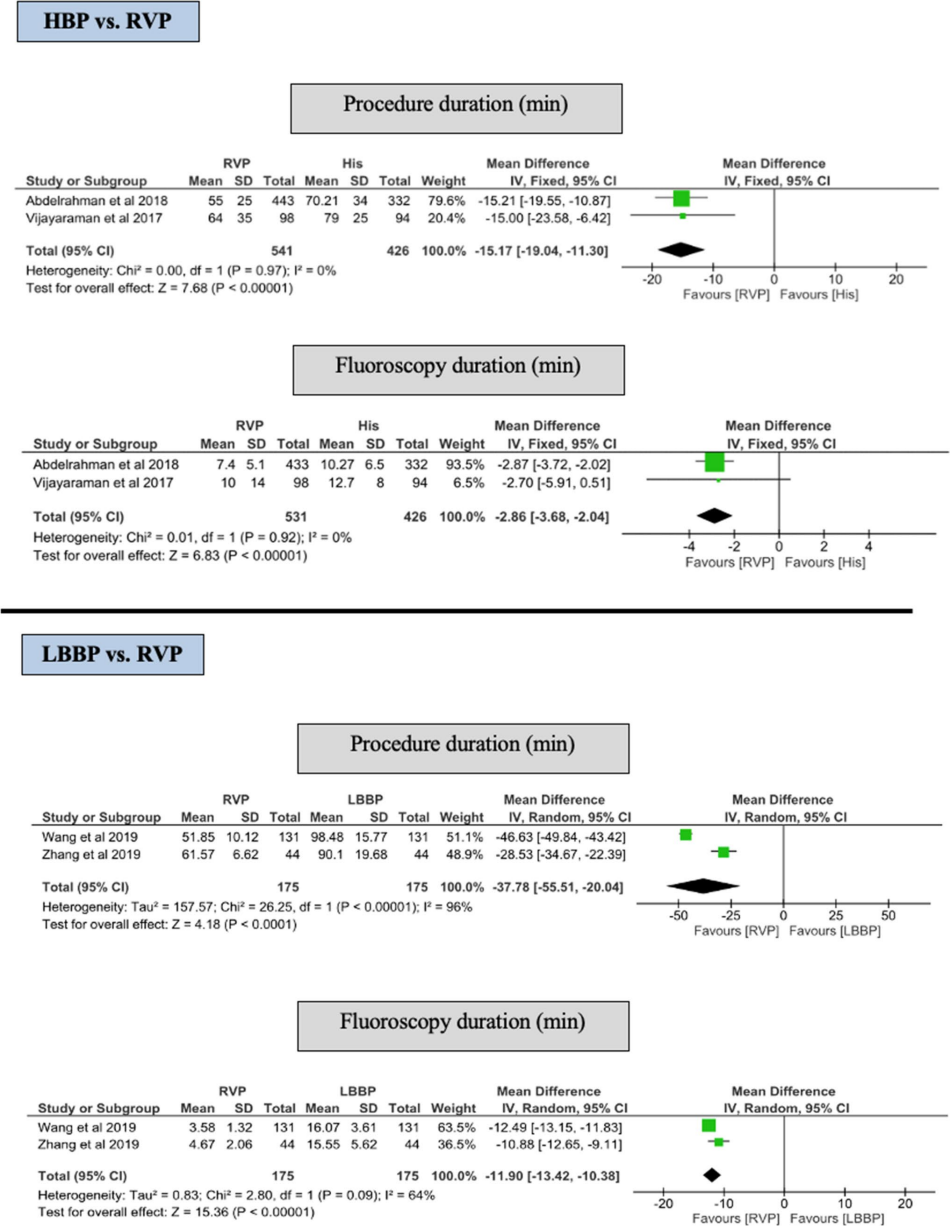
Forest plot of procedure and fluoroscopy among HBP and LBBP groups. *CI* confidence interval, *HBP* His-bundle pacing, *LBBP* left bundle branch pacing, *RR* risk reduction, *RVP* right ventricular pacing

HPB was associated with significantly longer procedure and fluoroscopy duration compared with RVP (MD of 15.17 min, 95% CI 11.30–19.04) vs. 2.86 min (95% CI 2.04–3.68) (*p* < 0.001), respectively (Fig. 5).

#### Lead revisions

During follow-up, ventricular lead revision was more frequently required in the HBP group compared with RVP group (RR: 5.83, 95% CI 2.17–15.70, *p* = 0.0005). The most common lead complication in HBP group was a progressive increase in the His capture threshold (14/426) followed by loss of capture (8/426) (Fig. 3).

### LBBP vs RVP

#### pQRS duration

Following implantation, the pQRS duration was shorter in the LBBP group (MD 5.6 ms, 95% CI −6.4 to 17.6 ms, *p* = 0.36) compared with the RVP group (51.0 ms, 95% CI 39.2–62.9 ms, *p* < 0.001) (Fig. 4). There was a significant interaction between LBBP and HBP concerning the effect on QRS duration (*p* for interaction < 0.001).

#### Capture threshold

**Fig. 6:**
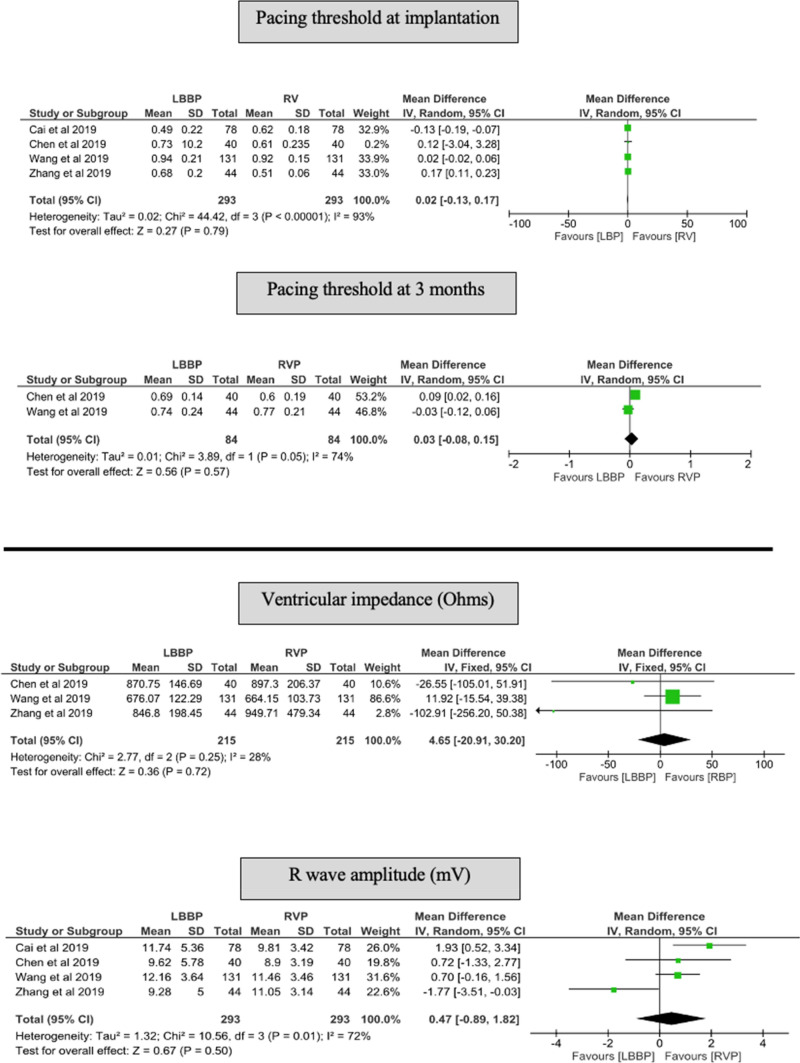
Forest plot of change in pacing threshold at implantation and at 3 months, ventricular impedance and R wave amplitude at implantation among LBBP and RVP groups. *CI* confidence interval, *LBBP* left bundle branch pacing, *RR* risk reduction, *RVP* right ventricular pacing

There was no difference in pacing capture thresholds in the LBBP group compared with RVP at the time of implantation (MD of 0.02 V, 95% CI − 0.13 to 0.17, *p* = 0.79), and at 3 months after implantation (MD of 0.03 V, 95% CI − 0.08 to 0.15, *p* = 0.57) (Fig. 6).

### Ventricular impedance and R wave amplitude at implantation

There was no difference in ventricular impedance and R wave amplitude in the LBBP group compared with RVP at the time of implantation (MD 4.65 Ohms, 95% CI − 20.91 to 30.20, *p* = 0.72) and (MD 0.47 mV, 95% CI − 0.89 to 1.82, *p* = 0.50), respectively (Fig. 6).

### Procedure and fluoroscopy duration

LBBP was associated with longer procedure and fluoroscopy durations compared to RVP (MD of 37.8 min, 95% CI 20.0–55.5) vs. (MD 11.9 min, 95% CI 10.4–13.4) (*p* = < 0.001) respectively (Fig. 5).

## Discussion

This systematic review and meta-analysis aimed to evaluate the existing evidence comparing HBP or LBBP with RVP as a primary pacing strategy. We found that compared with RVP, HBP was associated with a decrease in HFH rate, a decrease in the duration of the pQRS complex and a preservation of the LVEF, though this was at the expense of higher rates of lead revision and prolonged procedure and fluoroscopy duration. This study also found that LBBP was associated with a shorter pQRS complex duration compared with RVP, with no differences in pacing capture threshold at implantation and at 3 months, ventricular impedance and R wave amplitude at implantation, but an increase in procedure and fluoroscopy duration. We are not aware of other studies that have synthesized the comparative evidence for new pacing modalities including LBBP compared with RVP.

The risk of pacemaker-induced cardiomyopathy (PICM) is associated with a high burden of the pacing of the right ventricle [2, 6, 23]. PICM is generally defined as the deterioration of LVEF by at least 10%, resulting in LVEF < 50%, regardless of heart failure symptoms [2, 6]. Both BiVP and HBP may be effective in preventing or reversing PICM [17, 23–25]. Our study showed that HBP as an initial pacing strategy for patients requiring a permanent pacemaker is associated with a decrease in the risk of HFH, a reduction in pQRS complex duration, and a preservation of LVEF when compared with RVP. Of note, the difference in HFH rate might be due to different baseline LVEF and the cumulative rate of ventricular pacing (different cut-offs). Thus, this finding must be interpreted with caution, until the point that robust evidence is provided.

The duration of the pQRS complex is considered a strong predictor for the development of PICM regardless of pacing site, particularly when its duration is longer than 140 ms [2, 26]. A long pQRS duration contributes to electrical and mechanical desynchrony, which in turn leads to a deterioration of the LVEF [2–6]. Kim et al. found that a pQRS duration of > 140 ms was 95% sensitive for the detection of PICM while a pQRS duration of > 167 was 90% specific for the development of PICM [25]. In our analysis, both HBP and LBBP were associated with a significantly shorter pQRS duration compared with RVP. This finding may be explained by the physiological ventricle depolarization through His-Purkinje system which leads to a narrower pQRS duration and consequently reduces desynchrony [3, 16].

Others have performed meta-analyses that compare RVP with BiVP or HBP for patients with a normal or mildly reduced LVEF. One analysis compared HBP versus RVP on the measurements of left ventricular dimensions, LVEF, and symptom burden [10]. However, the studies included in this analysis reported outcomes among patients with LVEF > 35% who required permanent pacing because of AV block, and data on HFH were not available. The authors reported that LVEF remained preserved or increased with BiVP and HBP compared with RVP, with no observed effect on mortality. Our analysis included a larger cohort of patients and a new study for HBP [12]. Thus, our study reports data on mortality and HFH. Fernandes et al. performed a systematic review with network meta‐analysis comparing HBP, BiVP, and RVP as a primary pacing strategy for advanced AV conduction disease in patients with normal or mildly reduced EF (> 40%) [11]. This study found that HBP and BiVP were associated with a reduction in all‐cause mortality and HFH compared with RVP. HBP was superior to RVP with regards to LVEF deterioration, LV volumes, 6‐minute walk, and pQRS duration. However, this analysis only included patients with AV conduction disease and did not report data on lead revisions, new-onset AF and procedure duration or LBBP as an initial pacing strategy as we do in our study. Notably, in our study, we did not compare RVP with BiVP as an initial pacing strategy because of a small number of studies available in our study period.

Our meta-analysis reported data on mortality, HFH, LVEF changes, pQRS duration, AF occurrence, procedure duration and rates of lead revisions. Additionally, we included studies assessing LBBP as an initial pacing strategy compared with RVP. However, due to a small number of patients and outcomes evaluated in these studies, we only report data concerning pacing metrics and pQRS duration between LBBP and RVP. Our results indicate the potential advantages of conduction system pacing for patients requiring permanent pacing for bradycardia. Nonetheless, it is still not clear whether HPB might be beneficial in pure SND when RV stimulation is unnecessary. Additionally, raw patient data are needed to allow a better evaluation of patient characteristics. From mainly observational data, we found that HBP or LBBP are potentially superior to RVP as a first-line approach. However, there was a paucity of information about long-term efficacy and safety for these pacing-modalities.

This study has limitations. First, the majority of the studies included in the meta-analysis had small numbers of patients, different follow-up periods and predominantly were non-randomized single center studies. Of note, the reduction in HFH reported in our analysis was based on non-randomized studies comparing HBP performed in centres very experienced in HBP procedures with RVP performed in another centre, using RVP as routine [12, 17]. Second, the variation in the definitions between studies particularly those pertinent to exposure (e.g., pacing rate) or the outcome measures might have caused misclassification bias. As such, investigations were performed in patients with various pacemaker indications. In some, advanced AV block or at least the expectation of high cumulative pacing rates were mandatory to be included in the study, others preferred SND to avoid complications from potentially unreliable ventricular capture with HBP. Third, our analysis does not evaluate long-term performance of LBBP, with unknown feasibility of LBBP lead complication and extraction. Fourth, there was no available data on the clinical outcome of LBBP compared to RVP. When such data on long-term efficacy and safety are available, this may become a novel recommendation, given that current guidelines only recommend RVP as the pacing strategy for patients with preserved EF (1, 7). Fifth, there are no data available directly comparing clinical outcomes of HBP with LBBP which limits the ability to perform a network analysis between these strategies. Sixth, there were insufficient data to conduct a meaningful comparison between HBP or LBBP and RVP according to the position of the RV lead.

## Conclusion

Among patients undergoing de novo transvenous pacemaker implantation for bradyarrhythmia, an initial strategy of conduction system pacing compared with RVP was associated with shorter pQRS duration and preserved LVEF, but increased rates of lead revisions. LBBP compared with RVP was associated with a significant shorter pQRS duration with no difference in pacing metrics. Well conducted and robust randomised controlled comparative studies are needed to prove clinical outcome benefits from conduction system pacing.

### Supplementary Information

Below is the link to the electronic supplementary material.

Supplementary file1 (DOCX 31 KB)

## Data Availability

The data underlying this article are available in the article and in its online supplementary material.
