# Supplementary material for: Outcomes of conduction system pacing compared to right ventricular pacing as a primary strategy for treating bradyarrhythmia: systematic review and meta-analysis
Source: Clin Res Cardiol. 2021 Aug 19;111(11):1198–209. doi: 10.1007/s00392-021-01927-7 (PMC9622534; doi:10.1007/s00392-021-01927-7)
Supplement: Supplementary file 1 — Supplementary file1 (DOCX 31 KB) [file 392_2021_1927_MOESM1_ESM.docx]

**Supplementary material**

**Table S1.** Search strategy and Medical subject heading (MeSH) terms

| **Date of search** | | 17 November 2020 | |
| --- | --- | --- | --- |
| **Interface** | | Ovid | |
| **Databases** | | Embase 1996 to 2020 Week 46  Ovid MEDLINE(R) 1996 to November Week 1 2020 | |
| **Research question** | | For adult patients with bradycardia who require permanent pacing, what is the preferred primary pacing strategy?   - Right ventricular pacing (RVP) vs. His bundle pacing (HBP) - RVP vs. left bundle branch pacing (LBBP) | |
| **Population** | | Adults ≥ 18 years old, with bradycardia requiring permanent pacemaker | |
| **Intervention** | | Primary permanent pacing for bradycardia indication via RVP | |
| **Comparison** | | Primary permanent pacing for bradycardia indication via HBP, or  Primary permanent pacing for bradycardia indication via LBBP | |
| **Outcomes** | | Mortality  Heart failure hospitalisation  Change in left ventricular ejection fraction (LVEF) after pacing  New onset of atrial fibrillation (AF)  Paced QRS complex duration  Procedure duration  Lead revisions  Pacing threshold | |
| **Study type** | | Randomised controlled trials  Controlled observational trials | |
| **Timing** | | Full text publication between 01 January 2013 and 17 November 2020 | |
| **Embase** 1996 to 2020 Week 46 | | | |
| 1 | exp *heart block/ or sick sinus syndrome/ | | 16681 |
| 2 | (heart block or Auriculo-Ventricular Dissociation or A-V Dissociation or AV Dissociation or AV block or A-V block or Bundle-branch block or Mobitz or wenchebach$ or LBBB or AVB or RBBB or BBB or Cardiac block or ventriculoatrial block or ventricular block or atrial-ventricular block or atrio-ventricular block or atrioventricular block or Left anterior fascicular block or LAFB or interatrial block or aIAB or IAB or sinoatrial block or cardiac conduction block or heart conduction block or NOP-LBBB).ab,kw,ti. | | 54571 |
| 3 | 1 or 2 | | 61801 |
| 4 | dual chamber pacemaker/ or heart pacing/ or heart atrium pacing/ or heart ventricle pacing/ or leadless pacemaker/ or implantable cardiac monitor/ | | 35333 |
| 5 | (((Dual chamber or double-chamber or physiologic$) and (pacing or pacemaker$ or pace-maker$ or paced)) or DDDR or DDD$ or DCP$ or DOO$ or DVI$ or DDI$ or AV pac$ or A-V pac$ or atrioventricular pac$ or atrio-ventricular pac$ or ((rv or right ventricular or right ventricle or apical) and (pacing or pacemaker$ or pace-maker$ or paced)) or RVP).ab,kw,ti. | | 70891 |
| 6 | 4 or 5 | | 99720 |
| 7 | 3 and 6 | | 7658 |
| 8 | cardiac resynchronization therapy/ or cardiac resynchronization therapy device/ | | 29741 |
| 9 | (Cardiac resynchronization therapy or Cardiac resynchronisation therapy or CRT or ((Biventricular or biv) and (pacing or pacemaker$ or pacemaker$ or paced)) or bivp).ab,kw,ti. | | 40767 |
| 10 | His bundle/ | | 2712 |
| 11 | (pacing or pacemaker$ or pace-maker$ or paced).ab,kw,ti. | | 81339 |
| 12 | 10 and 11 | | 1245 |
| 13 | (((His-bundle or HB or bundle of his) and (pacing or pacemaker$ or pace-maker$ or paced)) or HBP).ab,kw,ti. | | 4793 |
| 14 | 8 or 9 or 12 or 13 | | 59993 |
| 15 | 7 and 14 | | 2169 |
| 16 | limit 15 to (english language and yr="2013 -Current") | | 1284 |
| 17 | 16 not ((exp animal/ or nonhuman/) not exp human/) | | 1229 |
| 18 | limit 17 to conference abstract status | | 657 |
| 19 | 17 not 18 | | 572 |
| **Ovid MEDLINE(R)**1996 to November Week 1 2020 | | | |
| 1 | exp Heart Block/ or Atrioventricular Block/ or Sick Sinus Syndrome/ | | 12177 |
| 2 | (heart block or Auriculo-Ventricular Dissociation or A-V Dissociation or AV Dissociation or AV block or A-V block or Bundle-branch block or Mobitz or wenchebach$ or LBBB or AVB or RBBB or BBB or Cardiac block or ventriculoatrial block or ventricular block or atrial-ventricular block or atrio-ventricular block or atrioventricular block or Left anterior fascicular block or LAFB or interatrial block or aIAB or IAB or sinoatrial block or cardiac conduction block or heart conduction block or NOP-LBBB).ab,kw,ti. | | 26612 |
| 3 | 1 OR 2 | | 31560 |
| 4 | Pacemaker, Artificial/ or Cardiac Pacing, Artificial/ or Electrocardiography, Ambulatory/ | | 29689 |
| 5 | (((Dual chamber or double-chamber or physiologic$) and (pacing or pacemaker$ or pace-maker$ or paced)) or DDDR or DDD$ or DCP$ or DOO$ or DVI$ or DDI$ or AV pac$ or A-V pac$ or atrioventricular pac$ or atrio-ventricular pac$ or ((rv or right ventricular or right ventricle or apical) and (pacing or pacemaker$ or pace-maker$ or paced)) or RVP).ab,kw,ti. | | 35316 |
| 6 | 4 or 5 | | 60366 |
| 7 | 3 and 6 | | 5874 |
| 8 | cardiac resynchronization therapy/ or cardiac resynchronization therapy device/ | | 4830 |
| 9 | (Cardiac resynchronization therapy or Cardiac resynchronisation therapy or CRT or ((Biventricular or biv) and (pacing or pacemaker$ or pacemaker$ or paced)) or bivp).ab,kw,ti. | | 16382 |
| 10 | His bundle/ | | 1159 |
| 11 | (pacing or pacemaker$ or pace-maker$ or paced).ab,kw,ti. | | 41752 |
| 12 | 10 and 11 | | 464 |
| 13 | (((His-bundle or HB or bundle of his) and (pacing or pacemaker$ or pace-maker$ or paced)) or HBP).ab,kw,ti. | | 2238 |
| 14 | 8 or 9 or 12 or 13 | | 19670 |
| 15 | 7 and 14 | | 1103 |
| 16 | limit 15 to (english language and yr="2013 -Current") | | 388 |
| 17 | 16 not (exp animals/ not exp humans/) | | 372 |
| 18 | limit 17 to case reports | | 73 |
| 19 | limit 17 to congress | | 1 |
| 20 | limit 17 to editorial | | 13 |
| 21 | limit 17 to comment | | 19 |
| 22 | 18 or 19 or 20 or 21 | | 96 |
| 23 | 17 not 22 | | 276 |

**Table S2.** Cochrane Risk of Bias Assessment for randomised trials

| Criteria/Study | Pastore et al. 2016 | Vijayaraman et al. 2017 | Abdelrahman et al. 2018 | Chen et al. 2019 | Cai et al. 2019 |
| --- | --- | --- | --- | --- | --- |
| Representativeness of the exposed cohort | 1 | 1 | 1 | 1 | 1 |
| Selection of the non-exposed Cohort | 1 | 1 | 1 | 1 | 1 |
| Ascertainment of exposure | 1 | 1 | 1 | 1 | 1 |
| Demonstration that outcome of interest was not present at start of study | 1 | 1 | 1 | 1 | 1 |
| Controlling for most important factor | 1 | 1 | 1 | 1 | 1 |
| Controlling for other factors | 0 | 0 | 0 | 0 | 0 |
| Assessment of Outcome | 1 | 0 | 0 | 0 | 0 |
| Follow-up long enough for outcomes to occur | 1 | 1 | 1 | 0 | 0 |
| Adequacy of follow up of cohorts (i.e., losses of follow up) | 1 | 1 | 1 | 0 | 1 |

**Table S3**. Newcastle-Ottawa Scale assessment for Cohort studies

| **Criteria** | Kronborg et al. 2014 | Wang et al. 2019 | Zhang et al. 2019 |
| --- | --- | --- | --- |
| Adequate randomisation sequence | Yes | No | No |
| Adequate concealment of allocation | Yes | No | No |
| Adequate blinding of study participants and personnel to participants’ allocated intervention | Yes | No | No |
| Adequate blinding of outcome assessors to participants’ allocated intervention | Yes | No | No |
| Adequate management of incomplete data | Yes | Yes | Yes |
| Absence of selective outcome reporting | Yes | Yes | Yes |
| Absence of other potential sources of bias | Yes | Yes | Yes |
